# The complete chloroplast genome of *Callicarpa integerrima* var. *chinensis* (Lamiaceae) from Guilin, China, a potential medicinal plant

**DOI:** 10.1080/23802359.2021.1969696

**Published:** 2021-08-27

**Authors:** Chunjiao Gu, Qiang Zhang, Deng Zhang, Yancheng Zhang, Junsong Liang

**Affiliations:** a College of Pharmacy, Guilin Medical University, Guilin, China; b Guangxi Institute of Botany, The Chinese Academy of Sciences, Guilin, China

**Keywords:** *Callicarpa integerrima* var. *chinensis*, chloroplast genome, phylogenetic relationship

## Abstract

*Callicarpa integerrima* var. *chinensis* is one of the traditional medical herbs which has the potential for multiple diseases’ treatment. In this study, the complete chloroplast genome sequence of *C. integerrima* var. *chinensis* was sequenced and assembled. A typical quadripartite structure was observed in the chloroplast genome of *C. integerrima* var. *chinensis* which was 154179 bp in length, including a pair of inverted repeat (IR) regions (each 51396 bp) separated by a large single-copy region (LSC) of 84927 bp and a small single-copy region (SSC) of 17856 bp, and the overall GC contents of the chloroplast genome was 38.08%. Additionally, we annotated 132 genes, including 86 protein-coding genes, 38 tRNA genes, and 8 rRNA genes. Phylogenetic analysis was adopted which confirmed the position of *C. integerrima* var. *chinensis* was close to the congeneric *C. formosana and C. siongsaiensis.*

*Callicarpa integerrima* var. *chinensis* (C. Pei) S. L. Chen is a herbaceous plant Lamiaceae (Chen and Gilbert 1994). It is widely distributed in Guangdong, Guangxi, and Hubei provinces of China and grows at the edge of forests, valleys and streams at an elevation of 200–1500 meters above sea level (Hu et al. 2013). In traditional Chinese medicine, preparations of this plant have been used for the treatment of bleeding, the common cold, cough, arthritis, and diarrhea (Zhu et al. 2012). Due to its high medicinal value, research has focused on the pharmacological action and mechanisms associated with these actions(Huang et al. 2014). However, there have been no genetic marker or plastid genome studies of *C. integerrima* var. *chinensis*. Here, we report and characteriz the complete plastid genome sequence of *C. integerrima* var. *chinensis* to contribute to its conservation and utilization.

Fresh leaves of *C. integerrima* var. *chinensis* were collected from the Guangxi Institute of Botany, the Chinese Academy of Sciences, Guilin, China (latitude: 25.0677; longitude:110.3037). Voucher specimen of *C. integerrima* var. *chinensis* was deposited at the herbarium of College of Pharmacy, Guilin Medical University (contact person: Junsong Liang, email: ljsylu@163.com) under the voucher number HCP-LJS-202104001). The total genomic DNA was extracted from fresh leaves of the sample using a CTAB method (Doyle and Doyle 1987) and was then sequenced using the Illumina Hiseq 2000 platform. Raw data were filtered using PRINSEQ lite Ver0.20.4 to obtain clean reads (5 GB) that were assembled with NOVOPlasty2.7.2. The de novo assembly was accomplished by GetOrganelle (Jin et al. 2020) using default settings. The assembled chloroplast genome was annotated by a combination of CPGAVAS2 (Shi et al. 2019) and GeSeq (Tillich et al. 2017). All genes we manually confirmed and adjusted using MEGA5.1 Beta2. The genome sequence and annotations were submitted to the NCBI database under accession number MW788028.

The complete chloroplast genome of *C. integerrima* var. *chinensis* is 154,179 bp in length, showing a typical quadripartite structure with a large single-copy region (LSC, 84,927 bp), a small single-copy region (SSC, 17,856 bp) and two inverted repeat regions (IRa and IRb, 51,396 bp). The GC content of the chloroplast genome is 38.08%. Annotation results showed the presence of 132 unique genes including 86 protein-coding, 38 tRNA and 8 rRNA genes. Seven protein-coding, seven tRNA and all four rRNA genes were duplicated in the IR regions. The chloroplast genome structure, gene order, GC content and codon usage are similar to typical angiosperm chloroplast genomes (Qian et al. 2013).

To confirm the phylogenetic position of *C. integerrima* var. *chinensis*, complete chloroplast genome sequences of this taxon and 17 other species selected from the Lamiaceae and one outgroup were used to construct phylogenetic tree. *Vitex rotundifolia* was selected as the outgroup of the phylogenetic tree. This study also used 58 protein-coding genes to conduct a ML analysis under the GTR + R + I model with 1000 bootstrap replicates. The phylogenetic tree fully resolved *C. integerrima* var. *chinensis* in a clade with *C. formosana and C. siongsaiensis* (Figure 1). By comparison, the results are consistent with Chen's. The complete chloroplast genome of *C. integerrima* var. *chinensis* reported here contributes to future studies on population genetics, evolutionary, and conservation biology in the Lamiaceae.

**Figure 1.**
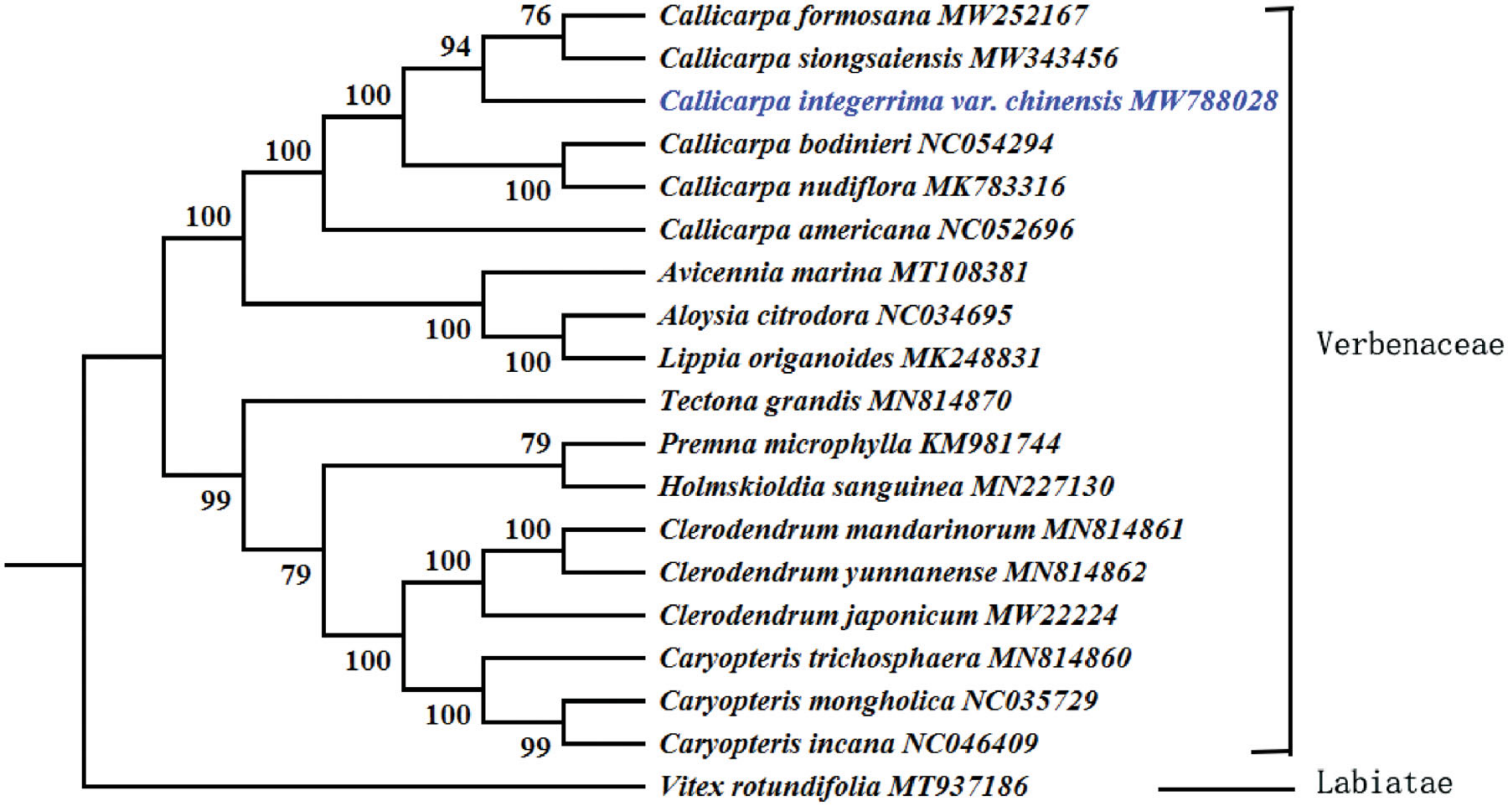
Phylogenetic tree construction using maximum likelihood based on 58 protein-coding genes from the complete chloroplast genome of *Callicarpa integerrima* var. *chinensis* and other 17 species within Verbenaceae and one outgroup species (*Vitex rotundifolia*). Numbers at the branches represent the bootstrap support values.

## Data Availability

The genome sequence data that support the findings of this study are openly available in GenBank of NCBI at (https://www.ncbi.nlm.nih.gov/) under the accession no. MW788028. The associated BioProject, SRA, and Bio-Sample numbers are PRJNA721567, SRR14235772, and SAMN18721261, respectively. Tree file of 19 species and genes for phylogenetic analysis were deposited at Figshare: https://doi.org/10.6084/m9.figshare.15133920
